# Plasma Metabolite Profiles Following Consumption of Animal Protein and Soybean-Based Diet in Hypercholesterolemic Postmenopausal Women

**DOI:** 10.3390/metabo12030209

**Published:** 2022-02-25

**Authors:** Neil K. Huang, Nirupa R. Matthan, Gregory Matuszek, Alice H. Lichtenstein

**Affiliations:** 1 Cardiovascular Nutrition Laboratory, Jean Mayer USDA Human Nutrition Research Center on Aging, Tufts University, Boston, MA 02111, USA; neil.huang@tufts.edu (N.K.H.); nirupa.matthan@tufts.edu (N.R.M.); 2 Biostatistics and Data Management Unit, Jean Mayer USDA Human Nutrition Research Center on Aging, Tufts University, Boston, MA 02111, USA; gregory.matuszek@tufts.edu

**Keywords:** animal protein, soybean protein, dietary biomarkers, metabolomics, clinical trials

## Abstract

Subjective reporting of food intake can be unreliable. No objective method is available to distinguish between diets differing in protein type. To address this gap, a secondary analysis of a randomized controlled cross-over feeding trial was conducted. Assessed were fasting plasma metabolite profiles and their associations with cardiometabolic risk factors (CMRFs). Hypercholesterolemic post-menopausal women (N = 11) were provided with diets containing predominantly animal protein (AP) and soy protein (SP). Untargeted metabolomics were used to determine the plasma metabolite profiles at the end of each diet phase. Concentrations of identified metabolites (N = 829) were compared using paired *t*-tests adjusted for false discovery rate, partial least square-discrimination analysis (PLS-DA) and receiver operating characteristics (ROC). Among the identified metabolites, 58 differed significantly between the AP and SP diets; the majority were phospholipids (*n* = 36), then amino acids (*n* = 10), xenobiotics (*n* = 7), vitamin/vitamin-related (*n* = 3) and lipids (*n* = 2). Of the top 10 metabolites, amino acid-derived metabolites, phospholipids and xenobiotics comprised the main categories differing due to dietary protein type. ROC curves confirmed that the top 10 metabolites were potential discriminating biomarkers for AP- and SP-rich diets. In conclusion, amino acid-derived metabolites, phosphatidylethanolamine-derived metabolites and isoflavones were identified as potential metabolite biomarkers distinguishing between dietary protein type.

## 1. Introduction

Evidence from both observational and interventional studies suggests that diet quality affects cardiovascular disease risk factors and outcomes [1,2]. Currently, the available tools to assess dietary intake rely heavily on subjective estimates of food intake, such as diet records and food frequency questionnaires, introducing a level of impression [3]. Incomplete and dated food composition tables used to analyze the food intake data further limit the reliability of the assessments. To date, few objective biomarkers are available to assess diet quality, fueling efforts to identify additional measures. 

Metabolomics is a promising tool that quantifies small molecules present in biological systems. Several studies suggest that metabolic profiling can distinguish among foods, groups of foods or dietary patterns [4,5,6,7]. Plasma and urine metabolites, such as alkylresorcinols, carnosine and 3-acylcarnitines, are established as biomarkers for dietary fiber [8], meat and fish [9], respectively. In the PREvención con DIeta MEDiterránea (PREDIMED) trial, an untargeted metabolomic approach was used to detect the differences in plasma metabolites between an animal and plant-based protein diet [10]. The findings indicated that C20:4 carnitine and dimethylglycine were negatively associated, while allantoin, C14:0 sphingomyelin, C38:7 phosphatidyl-ethanolamine plasmalogen, metronidazole, gamma aminobutyric acid and N-methylnicotinate were positively associated with animal protein intake [10]. Plasma metabolites in a cohort living in Japan found that hydroxyproline, 3-methylhistidine, aromatic amino acids, beta-alanine, carnitine and 2-aminobutyrate were positively associated with animal protein intake [11]. These studies identified different clusters of animal protein associated metabolites [4,9,10,11], with amino acids being the main metabolites that differed between dietary animal and plant proteins [12].

To date, few studies have compared the metabolite profiles of diets enriched in animal and soybean proteins during the conditions of a randomized controlled cross-over feeding trial. To address this limitation, we conducted a secondary analysis from a randomized clinical trial that compared diets containing predominantly soybean or animal protein on cardiovascular disease risk factors in hypercholesterolemic post-menopausal women. Untargeted metabolomic analysis was used to compare the plasma metabolite profiles after the participants consumed each of the two diets. We hypothesized that the fasting plasma metabolite profiles would differ based on the major protein source.

## 2. Results

### 2.1. Baseline Characteristics of Study Participants

The participants were post-menopausal women, aged 65 ± 6 y, with body mass index 27 ± 3 kg/m^2^ (Table 1). The subjects were recruited to have a low-density lipoprotein-cholesterol (LDL-C) concentration ≥ 3.4 mmol/L. 

### 2.2. Metabolomic Profiling

Of the identified plasma metabolites, after FDR adjustment for multiple testing, 7% (58 of 829) differed significantly at the end of the animal protein (AP) and soybean protein (SP) diet phases (Supplementary Table S1). Of those, 62% were phospholipids (*n* = 36), 17% were amino acids (*n* = 10), 12% were xenobiotics (*n* = 7), 5% were vitamin or vitamin-related (*n* = 3) and 3% were lipids (*n* = 2). Of the top 25 plasma metabolites that differed between the two diets, 64% were higher in the AP diet. These included phospholipids (*n* = 12), amino acids (*n* = 3) and xenobiotics (*n* = 1) (Table 2). The plasma concentrations of 3-methylhistidine, N-methylhistidine, carnitine, docosahexanoic acid, creatinine, 4-pyridoxic acid and D-pantothenic acid were higher after the participants consumed the AP compared to the SP diet. In contrast, six of the top 25 metabolites, daidzein 4-sulfate, daidzein, genistein, liquiritigenin, 4-hydroxymandelonitrile, ornithine and N-α-acetyl-L-ornithine, were higher after the participants consumed the SP compared to the AP diet (Supplementary Table S1). Additional information regarding the platform, InChI key, electrospray ionization mode, m/z values and retention time is summarized in Supplementary Table S2.

On the basis of a partial least squares discriminant analysis (PLS-DA), the first and second components explained 11.8% and 13.8% of the variance, respectively, between the AP and SP diets (Supplementary Figure S1). The cutoff threshold for the variable importance projection (VIP) score in this study was set at 1.0, and the top 10 metabolites were selected to reduce the total number of features to only those most highly associated with differences in dietary protein (Table 3). The VIP analysis indicated that 3-methylhistidine, 3-aminotyrosine, β-alanine, phosphatidylethanolamine (PE) O-37:5 (PE O-17:1_20:4), PE P-36:5 (or PE O-36:6), PE O-38:6 (PE O-18:1_20:5), PE P-34:2 or PE O-34:3, 3 isoflavones, N-α-acetyl-L-ornithine, 4-methylcatechol, liquiritigenin and 4-pyridoxate had scores greater than 1.5 (Table 3). A full list of the metabolites with VIP scores greater than 1.0, a well-established cutoff threshold, is presented in the Supplementary Table S3.

### 2.3. Area under the Curve-Receiving Operating Characteristics (AUC-ROC Curve) for Biomarker Analysis

The AUC-ROC curve was used to assess potential distinguishing biomarkers for the AP or SP diets. The data indicate that the AUC for the top 10 metabolites ranged from 0.85 to 1 (cutoff value: 0.8) (Table 4 and Supplementary Figure S2).

### 2.4. Enrichment and Network Analyses

**Figure 1:**
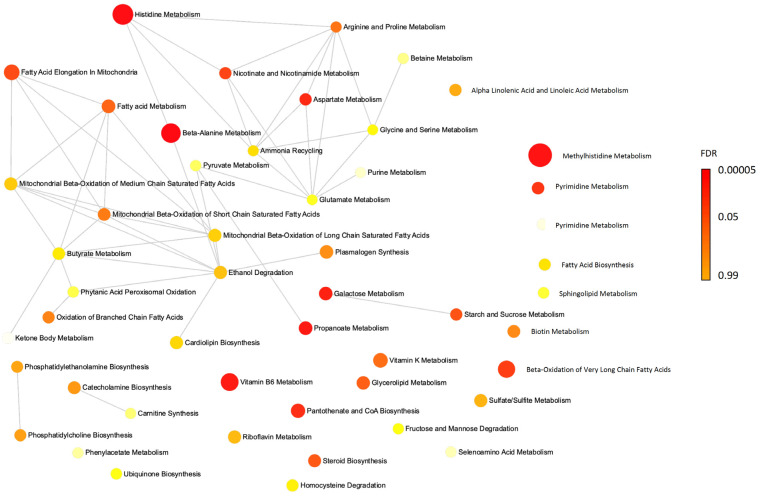
Network analysis for the plasma metabolites. Active pathways adjusted for FDR (<0.05) in participants received animal protein diet, compared to those in soybean protein.

Enrichment and network analyses indicated that the plasma metabolites (with FDR less than 0.05) that were higher after the participants consumed the AP diet are involved in several metabolic pathways (Supplementary Figure S3), including beta-alanine metabolism, histidine metabolism, methylhistidine metabolism, propanoate metabolism, vitamin B6 metabolism, galactose metabolism and aspartate metabolism (Table 5). A network analysis identified biological connections between these metabolites (Figure 1).

### 2.5. Correlations between Top 10 Metabolites and Cardiometabolic Risk Factors

The parent trial was specifically designed to compare the independent effects of different types of dietary protein. To avoid potential confounding by other variables to the greatest extent possible, the macronutrient, cholesterol and fiber, as well as fatty acid profiles of the diets, which would normally differ based on the predominant sources of protein, animal or plant, were adjusted to be similar. As the parent study reported, the effect of protein source, per se, had little effect on plasma lipids and lipoprotein concentrations (Supplementary Table S4) [13]. In the present analysis, there were no significant associations between metabolite concentrations and cardiometabolic risk factors (CMRFs) after the participants consumed the AP diet (Supplementary Table S5). There were significant associations between 3 phosphatidylethanolamines (PE) and CMRFs after the participants consumed the SP diet. These included plasma PE O-37:5 (PE O-17:1_20:4), which was positively correlated with plasma total cholesterol and low-density lipoprotein-cholesterol (LDL-C) concentration, and plasma PE O-38:6 (PE O-18:1_20:5) and PE P-36:5 (or PE O-36:6), which were positively correlated with high-density lipoprotein cholesterol (HDL-C) and apoprotein A1 concentrations. Plasma PE O-38:6 (PE O-18:1_20:5) was also negatively correlated with plasma triglyceride (TG) and very low-density lipoprotein-cholesterol (VLDL-C) concentrations (Supplementary Table S5). 

## 3. Discussion

This is the first study, using a randomized controlled cross-over feeding trial, to compare the effect of diets differing in the primary source of protein, AP or SP, on plasma metabolite profiles. Overall, based on the VIP score of each metabolite, the major differences were observed for some metabolites in the classes of PE and were amino acid-related. The scores were higher after the participants consumed the AP rather than the SP diet. Consistent with the constituent component of soybeans, ornithine-related and isoflavones metabolites were higher after the participants consumed the SP rather than the AP diet. A potentially favorable clinical biomarker identified after the participants consumed the SP diet was plasma PE O-38:6 (PE O-18:1_20:5), which was negatively correlated with the plasma TG and VLDL-C concentrations and positively correlated with plasma HDL-C and apoA1 concentrations.

Prior reports have identified some metabolite biomarkers for different types of dietary protein [14,15]. The European Prospective Investigation into Cancer and Nutrition (EPIC) study identified 3-methylhistidine as a potential biomarker for dietary poultry and some fish species [9]. Three-methylhistidine is stored in skeletal muscle. Carnosine, a dipeptide also present at high concentrations in muscle tissue, is composed of β-alanine and histidine. Our finding of higher concentrations of 3-methylhistidine and β-alanine after the participants consumed the AP compared to SP diet, during a controlled dietary feeding protocol, is consistent with that observed in two observational studies reporting positive correlations between these two amino acid-related metabolites and meat intake [11,14], in addition to EPIC. Our study design precluded an assessment of the biomarkers that distinguished among red meat, poultry or fish consumption. However, the network analysis indicated that the pathways for β-alanine, histidine and 3-methylhistidine metabolism were all significantly upregulated in the participants at the end of the AP relative to the SP diet phase, supporting the potential value of these amino acids as biomarkers for protein type.

In the current study, lipidomic analyses indicated that the majority of the metabolites that differed in response to the two diets belonged to the phospholipid class. Three of them, PE O-37:5 (PE O-17:1_20:4), PE P-36:5 (or PE O-36:6) and PE O-38:6 (PE O-18:1_20:5), were significantly higher after the participants consumed the AP compared to the SP diet. Similar differences between diets high in animal compared to plant proteins have been reported in one [15], but not other, observational studies [16,17,18]. The relations between individual phospholipid species and dietary protein type are complex, and the disparities observed among studies may be due to differences in the platforms used to measure the metabolites, methods used to assess dietary protein type and specific types of animal and plant proteins consumed [11,15,16,17,18].

The plasma concentrations of N-α-acetyl-L-ornithine and 3-aminotyrosine were significantly higher after the participants consumed the SP compared to the AP diet. From a protein quality perspective, SP is complete in terms of its amino acid profile, and particularly rich in phenylalanine, glutamate and arginine. These three amino acids are precursors of ornithine, and thus provide substrates for the urea cycle, arginine synthesis and aromatic amino acid metabolism. It has previously been reported that the L-ornithine supplement activated the urea cycle by increasing ornithine, necessary to transport amino groups out of mitochondria to the cytoplasm for subsequent urinary excretion [19]. These data suggest that N-α-acetyl-L-ornithine may be a plasma biomarker for dietary SP. However, this premise is tempered because 3-aminotyrosine is an intermediate in aromatic amino acid metabolism and can be formed via tyrosine metabolism. It has been proposed that phenylalanine is converted to tyrosine and involved in tyrosine metabolism or tyrosine modification [20].

The plasma concentrations of isoflavones, daidzein 4-sulfate, daidzein and genistein were all significantly higher after the participants consumed the SP compared to the AP diet. These findings, documented previously, reflect the abundance of these compounds in SP [21,22,23]. The SP diet contained 15.4 mg of daidzein and 47.9 mg genistein per 1000 kcal, whereas the AP diet contained 0.6 mg of daidzein and 9.8 mg genistein per 1000 kcal [13]. However, the microbiome can modify the isoflavones prior to absorption, hence making the levels somewhat tenuous [24]. Hence, further studies are warranted to determine whether isoflavones are appropriate plasma biomarkers for soybean consumption.

Of the PE metabolites identified that differed between the AP and SP diets, PE O-38:6 (PE O-18:1_20:5) was the most closely associated with CMRFs. PEs have many cellular functions, such as lipid homeostasis, serving substrates for post-translational modification [25]. More specifically, they play a crucial role in VLDL-C assembly and secretion, and the compositions of PEs and other phospholipids in nascent plasma VLDL-C are highly associated with that in the liver [25,26,27]. Additionally, once released into circulation, PEs are rapidly removed from VLDL-C as the particles are delipidated [26]. In contrast to prior reports, the present study plasma PE O-38:6 (PE O-18:1_20:5) was negatively associated with VLDL-C concentrations [25,26,27]. This discrepancy may be due to differences in the blood fraction of the PEs that was measured, plasma as in the current study or VLDL-C particles. Data from a global CTP:phosphoethanolamine cytidylyltransferase knockout mouse model (Pcyt2+/−) indicate that a lack of PE globally did not alter hepatic VLDL-C secretion. The plasma VLDL-C concentrations in Pcyt2+/− mice were higher compared to those in wide-type mice [27]. In contrast, hepatic Pcyt2+/− mice developed steatosis and had lower hepatic VLDL-C secretion [25]. This suggests that only hepatic PEs may have a positive association with plasma VLDL-C concentration, and PEs in circulation or from other tissues may have a negative association with VLDL-C. 

The associations observed between the plasma PEs and CMRFs after the participants consumed the SP diet suggest potential involvement in cholesterol, TG and lipoprotein metabolism, particularly for PE O-38:6 (PE O-18:1_20:5). Previous studies indicated that plasma omega-3 fatty acids have been positively associated with increased HDL-C, lowered plasma TG, VLDL-C and apolipoprotein B [28,29]. Given the relatively high in docosahexaenoic acid and eicosapentaenoic acid (EPA)-content in PE O-38:6 (PE O-18:1_20:5) in the participants consuming the SP diet, we postulate there is a synergistic effect of omega-3 fatty acids, and this PE species is resulting in this observation. 

A strength of this study is that the plasma samples and CMRF data were collected after the participants consumed both the AP and SP diets in a random order as part of a controlled cross-over trial. The diets were carefully designed to differ primarily in the type of protein and were matched for fiber, cholesterol, macronutrient distribution and fatty acid profile. The cross-over design minimized confounding due to inter-individual differences. A wide range of metabolites were measured by using a combination of three analytical platforms. As expected, the plasma isoflavone concentrations were the primary biomarkers identified after the consumption of the SP and confirm a high level of compliance with the study protocol by the study participants. A limitation of the study is that the AP diet contained a mixture of animal proteins (red meat, chicken, eggs and dairy products); hence, the effect of individual protein sources could not be determined. Although the samples were stored at −80 °C and never thawed, they were collected approximately 15 years ago; thus, we cannot rule out potential metabolite loss or degradation. Only the identified metabolites were assessed. Further research is needed to identify potential unknown metabolites that differed by dietary protein type. 

## 4. Materials and Methods

### 4.1. Study Participants and Design

The participants included in the current study (*n* = 11, all females) were randomly selected from a randomized controlled cross-over feeding trial (referred to as parent trial) [13]. Participant recruitment criteria included ≥ 50 y, LDL-C concentrations ≥ 3.36 mmol/L, postmenopausal status, no chronic illnesses and no treatment with lipid-lowering drugs. Some of the data, related to a different experimental question, have been reported previously [13]. Fasting plasma samples collected at the end of diet phase were stored at −80 °C and never thawed. The parent trial was registered at clinicaltrials.gov as NCT00175097 (last accessed on 6 January 2022).

### 4.2. Diet Intervention

Each dietary phase was 6 weeks in length, during which all food and beverages were provided to study participants. There was a washout period of at least 2 weeks between diet phases during, which participants ate their habitual diets. Both the AP and SP diets were composed of 55% total energy as carbohydrate, 17% protein and 28% fat. Half the protein in the SP diet consisted of foods made with whole soybeans (Supplementary Table S6) [13]. These included whole cooked organic soybeans (Westbrae Natural, Hain Food Group Inc., Uniondale, NY, USA), roasted soynuts (Solnuts Inc., Hudson, IA, USA), defatted soyflakes (Cargill Inc., Cedar Rapids, IA, USA), soya granules (Fearn Natural Foods, Mequon, WI, USA) and soynut butter (Health Trip Co., Concord, MA, USA). An equivalent amount of protein in the AP diet came from a mixture of red meat, chicken, eggs and dairy products. The cholesterol and fiber content of the diets were balanced to avoid confounding by these variables. Consistent with the aims of the parent study, the fatty acid profile was adjusted to be similar so that the major difference between the two experimental diets was the type of protein. All food and drink were provided, and body weight was maintained throughout the study period by adjusting energy intake, if necessary. The detailed protocol menu for AP and SP diets was published previously [13].

### 4.3. Untargeted Metabolomics

Three platforms for untargeted metabolomic analyses were performed by the West Coast Metabolomics Center at University of California, Davis using a combination of gas chromatography/time-of-flight mass spectrometry (GCTOF MS) and ultra-high pressure liquid chromatography/quadrupole time-of-flight tandem mass spectrometry (UHPLC-QTOF MS/MS) [30,31,32,33]. Internal standards and pooled samples were routinely all included in sample preparation for quality and quantity purposes. Pooled samples were used to ensure the data reliability and reproducibility when compounds were detected in both platforms. All the methods used in this study were published and validated [30,31,32].

#### 4.3.1. Primary Metabolites Extraction and Data Acquisition

The extraction protocol and methodology for the GCTOF MS analyses were previously reported [30,31]. BinBase database was used for compound identification.

Samples were measured on LECO Pegasus IV time of flight mass spectrometers (LECO Corporation, St. Joseph, MI, USA) [30]. An Rtx -5Sil MS column (30 m × 0.25 mm internal diameter, 0.25 μm film made of 95% dimethyl/5% diphenylpolysiloxane; Restek Corporation, Bellefonte, PA) was employed with helium as a carrier gas at flow rate of 1 mL/min. Sample injection volume was 0.5 μL with an injection condition of 25 splitless time into a multi-baffled glass liner. The injection temperatures were 50 °C ramped to 250 °C by 12 °C/sec. The oven temperature was 50 °C, held for 1 min, then increased to 330 °C at a rate of 20 °C/min and then held constant for 2 min. For quality assurance, automatic liner exchanges were applied after each set of 10 injections to minimize sample carryover for highly lipophilic compounds, and pooled and blank samples were included every 10 and 50 samples, respectively. 

#### 4.3.2. Complex Lipids and Biogenic Amines Extraction and Data Acquisition

The protocols for lipids and biogenic amines extractions were similar and described previously [32]. UHPLC-QTOF MS/MS was performed for lipidomics and biogenic amines [33]. Mass Hunter was used for compound identification.

Samples for lipidomics (complex lipid profiling) were measured on Agilent 6530 (R = 10,000 for positively charged compounds) and Agilent 6550 (R = 20,000 for negatively charged compounds) QTOF mass spectrometers (Agilent Technologies, Santa Clara, CA, USA) [33]. An UHPLC charged surface hybride C18 column (100 m × 2.1 mm internal diameter, 1.7 μm particles; Waters Corporation, Milford, MA) was employed at flow rate of 0.6 mL/min. Sample injection volume was 3 μL with an injection temperature at 4 °C. The mobile phase A (A) was a solvent containing acetonitrile:water (60:40; *v*/*v*) plus 10 mM ammonium formiate and 0.1% formic acid. Mobile phase B (B) contained isopropanol:acetonitrile (90:10; *v*/*v*) plus 10 mM ammonium formiate and 0.1% formic acid. The column temperature was 65 °C, and gradient was set as follows: 0 min 15% (B), 0–2 min 30% (B), 2–2.5 min 48% (B), 2.5–11 min 82% (B), 11–11.5 min 99% (B), 11.5–12 min 99% (B), 12–12.1 min 15% (B), 12.1–15 min 15% (B). For quality assurance, automatic liner exchanges were applied after each set of 10 injections to minimize sample carryover for highly lipophilic compounds, and pooled and blank samples were included every 10 and 50 samples, respectively.

Samples for biogenic amines analysis were measured on an Agilent 6530 (R = 10,000) QTOF mass spectrometer (Agilent Technologies, Santa Clara, CA, USA) [33]. An UPLC BEH Amide VanGuard pre-column (5 mm × 2.1 mm internal diameter, 1.7 μm; Waters Corporation, Milford, MA) and an UHPLC BEH Amide column (100 m × 2.1 mm internal diameter, 1.7 μm; Waters Corporation, Milford, MA, USA) were employed at flow rate of 0.4 mL/min. The columns were kept at 40 °C. Sample injection volume was 3 μL for electrospray ionization (ESI) (+) with an injection temperature at 4 °C. The mobile phase A (A) was a solvent containing ultrapure water plus 10 mM ammonium formiate, 0.125% formic acid and pH value was adjusted to 3. Mobile phase B (B) contained acetonitrile:ultrapure water (95:5; *v*/*v*) plus 10 mM ammonium formate, 0.125% formic acid and pH 3. The gradient was set as follows: 0 min 100% (B), 0–2 min 100% (B), 2–7 min 70% (B), 7.7–9 min 40% (B), 9.5–10.25 min 30% (B), 10.25–12.75 min 100% (B), 16.75 min 100% (B). The ESI capillary voltage was +4.5 kV, and collision energy was +45 eV for ESI (+). Precursor isolation width was set to 3 Da, and scan range was m/z 60–12,000 Da. For quality assurance, automatic liner exchanges were applied after each set of 10 injections to minimize sample carryover for highly lipophilic compounds, and pooled and blank samples were included every 10 and 50 samples, respectively.

### 4.4. Clinical Laboratory Measures

Plasma concentrations of total cholesterol, TG, HDL-C and LDL-C were measured at the end of each diet phase using a Hitachi 911 automated analyzer (Hitachi, Indianapolis, IN, USA) with the use of Roche Diagnostics reagents, as previously reported [13]. HDL-C subfractions were determined using a modified dextran sulfate–magnesium chloride method [34]. VLDL-C concentration was calculated using the following formula: total cholesterol (mmol/L)—(LDL-C + HDL-C). 

### 4.5. Statistical Analysis

To characterize the study participants at the end of study, we calculated means and standard deviations (SD) for continuous measures and proportions for categorical measures. 

For metabolomics data processing, a total of 3028 metabolites were identified using three platforms, in which 841 were known metabolites. The data for known metabolites were normalized by the pooled samples and were imputed with the minimum detectable values only for those values that were missing less than 80%, otherwise were excluded (12 known metabolites were excluded). This study focused on the known metabolites (829 metabolites), and the data were filtered by the interquartile range and were log-transformed prior to statistical analysis. 

In the primary analysis, paired *t*-test was used to assess the differences of the metabolites between AP and SP diets, and the Benjamini & Hochberg procedure was conducted to account for multiple comparisons (0.05/829, *p* < 6.03 × 10^−5^; FDR < 0.05). PLS-DA was then used to assess the importance of each metabolite using the VIP score, and the metabolites were identified and ranked from the highest to the lowest (cutoff: 1.0). The top 10 metabolites were then selected to reduce the total number of features to only those most strongly associated with differences in the sources of dietary protein. A receiver operating characteristic curve was then conducted as a sensitivity analysis to assess the accuracy of the potential biomarkers in this model, for which area under the curves and confidence intervals were computed (cutoff: 0.8). Lastly, the metabolites were mapped using metabolite set enrichment analysis to identify patterns and metabolic pathways associated with consumption of the AP or SP diet [35].

To further understand whether the plasma metabolites were associated with any of the cardiometabolic risk factors, we assessed the correlations between the top 10 metabolites and cardiometabolic risk factors separately by protein type using Pearson correlation coefficient. 

Analyses were conducted using R (version 3.6.3; Vienna, Austria) and MetaboAnalyst (version 5.0; https://www.metaboanalyst.ca/home.xhtml; last accessed on 18 November 2021) [36]. Statistical significance was defined as two-tailed α ≤ 0.05.

## 5. Conclusions

Identified were the metabolites that differed after the participants consumed the AP and SP diets, potentially representing biomarkers of the dietary protein type. Of the PE metabolites identified after the consumption of the SP diet, the plasma PE O-38:6 (PE O-18:1_20:5) was the most favorably associated with the plasma lipid profile. 

## Figures and Tables

**Table 1:** Baseline characteristics of the study participants.

Variables	Participants (N = 11)
Age, y	65 ± 6
Weight, kg	71 ± 12
Females (%)	100
Body Mass Index, kg/m^2^	27.3 ± 3.4
Total cholesterol, mmol/L	6.18 ± 0.62
VLDL-C, mmol/L	0.54 ± 0.20
LDL-C, mmol/L	3.96 ± 0.63
HDL-C, mmol/L	1.67 ± 0.38
Triacylglycerol, mmol/L	1.19 ± 0.44

All values were presented as mean ± SD. To convert values for total cholesterol, VLDL-C, LDL-C, HDL-C and triacylglycerol, multiply 38.67 and 88.54, respectively. HDL-C, high-density lipoprotein-cholesterol; LDL-C, low-density lipoprotein-cholesterol; VLDL-C, very low-density lipoprotein-cholesterol.

**Table 2:** Selected top 25 plasma metabolites.

Metabolites	Category	*t*-Statistics	FDR
Daidzein 4′-sulfate	Xenobiotics	−20.13	0.0000017
PE 38:4	Phospholipids	−9.516	0.0008065
PE 38:4 Isomer B	Phospholipids	−9.074	0.0008065
PE P-34:2 or PE O-34:3	Phospholipids	9.062	0.0008065
3-Methylhistidine	Amino acids	8.455	0.0011983
PC P-36:5 or PC O-36:6	Phospholipids	7.758	0.0019206
PE O-37:5 (PE O-17:1_20:4)	Phospholipids	7.614	0.0019206
N-α-Acetyl-L-ornithine	Amino acids	−7.491	0.0019206
N-Methylhistidine	Amino acids	7.491	0.0019206
PE P-36:4 or PE O-36:5	Phospholipids	7.025	0.0029885
PC P-38:6 or PC O-38:7	Phospholipids	6.740	0.0037446
PE O-38:6 (PE O-18:1_20:5)	Phospholipids	6.637	0.0037446
PE 36:4	Phospholipids	−6.628	0.0037446
3-Aminotyrosine	Amino acids	−6.466	0.0042649
PE 38:5 (PE 16:0_22:5)	Phospholipids	−6.387	0.0044006
(2R)-3-Hydroxyisovaleroylcarnitine	Amino acids	6.173	0.0053753
PE 36:1 (PE 18:0_18:1)	Phospholipids	−6.137	0.0053753
PE P-38:3 or PE O-38:4	Phospholipids	6.035	0.0057137
PC O-36:3	Phospholipids	6.007	0.0057137
PE P-38:6 or PE O-38:7	Phospholipids	5.747	0.0075186
PC P-34:1 or PC O-34:2	Phospholipids	5.729	0.0075186
PC 40:5 Isomer B	Phospholipids	−5.601	0.0085627
(3-Carboxypropyl)trimethylammonium	Xenobiotics	5.550	0.0088038
PC P-34:1 or PC O-34:2 Isomer A	Phospholipids	5.479	0.0093114
PC O-37:5	Phospholipids	5.394	0.0100850

The top 25 metabolites are presented. Benjamini & Hochberg procedure was used to adjust for multiple comparisons, and statistical significance was defined as FDR < 0.05. PC, phosphatidylcholine; PE, phosphatidylethanolamine.

**Table 3:** Top 10 metabolites with the highest variable importance in projection (VIP) ^1^ scores.

Metabolite	Pathway Involved	VIP Score ^1^
Daidzein 4′-sulfate	Xenobiotics	16.5
Genistein	Xenobiotics	7.22
Daidzein	Xenobiotics	7.16
3-Methylhistidine	Amino acid	4.57
N-α-Acetyl-L-ornithine	Amino acid	2.55
3-Aminotyrosine	Amino acid	2.54
PE O-37:5(PE O-17:1_20:4)	PE/lipid metabolism	2.49
PE P-36:5 or PE O-36:6	PE/lipid metabolism	2.03
PE O-38:6 (PE O-18:1_20:5)	PE/lipid metabolism	1.97
β-alanine	Amino acid	1.73

^1^ Variable importance in projection (VIP) score was calculated using partial least-squares discrimination analysis. This table shows top 10 plasma metabolites with highest VIP scores. PE, phosphatidylethanolamine.

**Table 4:** Area under the curve-receiver operating characteristics (AUC-ROC) curves for the top 10 plasma metabolites.

Metabolites	AUC	*p* Value
Daidzein 4’-sulfate	1	9.51 × 10^−12^
Genistein	0.99	2.44 × 10^−4^
Daidzein	0.97	6.09 × 10^−5^
3-Methylhistidine	0.96	6.92 × 10^−5^
PE O-37:5 (PE O-17:1_20:4)	0.93	3.07 × 10^−5^
PE O-38:6 (PE O-18:1_20:5)	0.91	6.47 × 10^−4^
N-α-Acetyl-L-ornithine	0.90	2.03 × 10^−3^
PE P-36:5 or PE O-36:6	0.89	1.19 × 10^−3^
3-Aminotyrosine	0.87	3.01 × 10^−3^
β-alanine	0.85	3.98 × 10^−3^

AUC-ROC curves were performed (cutoff: 0.8). AUC, area under the curve; PE, phosphatidylethanolamine.

**Table 5:** Active metabolic pathways in participants who received animal protein diet.

Pathway	*p-*Value	FDR
Beta-alanine metabolism	0.00000037	0.0000336
Histidine metabolism	0.00000143	0.0000652
Methylhistidine metabolism	0.0000154	0.000468
Propanoate metabolism	0.000147	0.00334
Vitamin B6 metabolism	0.00209	0.038
Galactose metabolism	0.00313	0.0426
Aspartate metabolism	0.00328	0.0426
Pantothenate and CoA biosynthesis	0.00539	0.0563
Pyrimidine metabolism	0.00582	0.0563
Beta oxidation of very long chain fatty acids	0.00619	0.0563

The Benjamini & Hochberg procedure was conducted to account for multiple comparisons, and statistical significance was defined as FDR < 0.05. FDR, false discovery rate.

## Data Availability

Duo to confidentiality agreements, the data that support the findings of this study are available from the corresponding authors upon reasonable request.

## Supplementary Materials

The following supporting information can be downloaded at: https://www.mdpi.com/article/10.3390/metabo12030209/s1, Supplementary Tables S1–S6; Supplementary Figures S1–S3.

## Abbreviations

AP	animal protein
CMRF	cardiometabolic risk factor
CVD	cardiovascular disease
DHA	docosahexaenoic acid
EPA	eicosapentaenoic acid
GCTOF MS	gas chromatography/time-of-flight mass spectrometry
HDL-C	high-density lipoprotein-cholesterol
LDL-C	low-density lipoprotein-cholesterol
UHPLC-QTOF MS/MS	ultra-high performance liquid chromatography-quadrupole time-of-flight tandem mass spectrometry
PC	phosphatidylcholine
PE	phosphatidylethanolamine
PLS-DA	partial least squares discriminant analysis
ROC curve	receiving operating characteristics curve
SP	soybean protein
TG	triglyceride
VIP	variable importance projection
VLDL-C	very low-density lipoprotein-cholesterol
