# Supplementary material for: Plasma Metabolite Profiles Following Consumption of Animal Protein and Soybean-Based Diet in Hypercholesterolemic Postmenopausal Women
Source: Metabolites. 2022 Feb 25;12(3):209. doi: 10.3390/metabo12030209 (PMC8952012; doi:10.3390/metabo12030209)
Supplement: Supplementary file 1 [file metabolites-12-00209-s001.zip › metabolites-1582176-supplementary.pdf]

Plasma metabolite profiles following consumption of animal protein and soybean-based diet in hypercholesterolemic postmenopausal women

Neil K. Huang,[1] Nirupa R. Mattha,[1] Gregory Matuszek,[2] Alice H. Lichtenstein[1,*]

[1]Cardiovascular Nutrition Laboratory, Jean Mayer USDA Human Nutrition Research Center on Aging, Tufts University, Boston, MA 02111 U.S.A.

[2]Biostatistics and Data Management Unit, Jean Mayer USDA Human Nutrition Research Center on Aging, Tufts University, Boston, MA 02111 U.S.A.

[*]Corresponding author: Alice H. Lichtenstein, D.Sc.,

Jean Mayer USDA Human Nutrition Research Center on Aging

Tufts University

711 Washington Street

Boston, MA 02111

Phone: (617)556-3127

Short title: Plasma biomarkers for protein diets

Journal Subject Terms: Dietary Composition, Dietary Biomarkers, Metabolomics, and Prevention/ Cardiovascular Disease

Supplemental Table S1. Plasma metabolites with FDR values < 0.05, comparison between animal protein and soybean protein diets

Metabolites	Category	*t*-statistics	FDR
Daidzein 4'-sulfate	Xenobiotics	-20.13	0.0000016703
PE 38:4	Phospholipids	-9.5164	0.00080652
PE 38:4 Isomer B	Phospholipids	-9.0735	0.00080652
PE P-34:2 or PE O-34:3	Phospholipids	9.0617	0.00080652
3-Methylhistidine	Amino acids	8.4554	0.0011983
PC P-36:5 or PC O-36:6	Phospholipids	7.7582	0.0019206
PE O-37:5 (PE O-17:1_20:4)	Phospholipids	7.6142	0.0019206
N-α-Acetyl-L-ornithine	Amino acids	-7.4912	0.0019206
N-Methylhistidine	Amino acids	7.4907	0.0019206
PE P-36:4 or PE O-36:5	Phospholipids	7.0251	0.0029885
PC P-38:6 or PC O-38:7	Phospholipids	6.7395	0.0037446
PE O-38:6 (PE O-18:1_20:5)	Phospholipids	6.6372	0.0037446
PE 36:4	Phospholipids	-6.6275	0.0037446
3-Aminotyrosine	Amino acids	-6.4657	0.0042649
PE 38:5 (PE 16:0_22:5)	Phospholipids	-6.3871	0.0044006
(2R)-3-Hydroxyisovaleroylcarnitine	Amino acids	6.1725	0.0053753
PE 36:1 (PE 18:0_18:1)	Phospholipids	-6.1367	0.0053753
PE P-38:3 or PE O-38:4	Phospholipids	6.0349	0.0057137
PC O-36:3	Phospholipids	6.0066	0.0057137
PE P-38:6 or PE O-38:7	Phospholipids	5.7474	0.0075186
PC P-34:1 or PC O-34:2	Phospholipids	5.7294	0.0075186
PC 40:5 Isomer B	Phospholipids	-5.6014	0.0085627
(3-Carboxypropyl) trimethylammonium	Xenobiotics	5.5495	0.0088038
PC P-34:1 or PC O-34:2 Isomer A	Phospholipids	5.4791	0.0093114
PC O-37:5	Phospholipids	5.3937	0.010085
Liquiritigenin	Xenobiotics	-5.271	0.011148
PC O-36:4	Phospholipids	5.2695	0.011148
PE P-40:6 or PE O-40:7	Phospholipids	5.0996	0.013747
L-Cysteine S-sulfate	Amino acids	5.0593	0.014079
Ornithine	Amino acids	-4.9536	0.015865
PC 36:5 Isomer C	Phospholipids	-4.933	0.015865
PE P-38:4 or PE O-38:5	Phospholipids	4.8565	0.016948
PE P-36:5 or PE O-36:6	Phospholipids	4.8467	0.016948
PC P-38:5 or PC O-38:6 Isomer A	Phospholipids	4.7115	0.020164
Daidzein	Xenobiotics	-4.6847	0.020281
Genistein	Xenobiotics	-4.6518	0.020281
PC P-36:4 or PC O-36:5	Phospholipids	4.6507	0.020281
Carnitine	Amino acids	4.6346	0.020281
4-Pyridoxate	Vitamin	4.6104	0.020504
PC 38:5 Isomer A	Phospholipids	-4.5197	0.02298
Docosahexanoic acid	Lipids	4.4926	0.023117
PC P-34:2 or PC O-34:3	Phospholipids	4.4843	0.023117

PC P-38:5 or PC O-38:6	Phospholipids	4.3119	0.029544
PC P-40:6 or PC O-40:7 Isomer B	Phospholipids	4.244	0.032136
PC 38:6 Isomer A	Phospholipids	-4.1152	0.038287
Ecgonine	Xenobiotics	4.1059	0.038287
D-Pantothenic acid	Vitamin	4.0053	0.044043
PE P-38:5 or PE O-38:6	Phospholipids	3.9828	0.044722
Beta-alanine	Amino acid	3.9371	0.046108
PE P-34:1 or PE O-34:2	Phospholipids	3.9091	0.046108
PE P-36:2 or PE O-36:3	Phospholipids	3.9057	0.046108
4-Hydroxymandelonitrile	Xenobiotics	-3.9029	0.046108
PE 36:2	Phospholipids	-3.9027	0.046108
PE P-40:7 or PE O-40:8	Phospholipids	3.827	0.049239
4-Methylcatechol	Amino acids	-3.8049	0.049239
Nudifloramide	Vitamin	3.8028	0.049239
TG 55:4 (TG 18:1_19:1_18:2)	Lipids	-3.8007	0.049239
PE P-36:1 or PE O-36:2	Phospholipids	3.7968	0.049239

The Benjamini and Hochberg procedure was conducted to account for multiple comparisons, and statistical significance was defined as FDR< 0.05. FDR, false discovery rate; PC, phosphatidylcholine; PE, phosphatidylethanolamine. TG, triglyceride.

Supplemental Table S2. The IUPAC International Chemical Identifier keys, electrospray ionization mode, m/z value and retention time for the metabolites in the Tables 2 and 3

Platform	Metabolites	In ChI key	ESI mode	m/z	RT
UHPLC-QTOF MS/MS	Daidzein 4'-sulfate	CQRHJEVIKOKQMO-UHFFFAOYSA-N	ESI (-)	333.0057	7.29
	PE 38:4	ANRKEHNWXKCXDB-BHFWLYLHSA-N	ESI (+)	768.557	5.68
	PE P-34:2 or PE O-34:3	LALFTIIBOJSVNP-OBEJFMRTSA-N	ESI (-)	698.5136	5.96
	3-Methylhistidine	JDHILDINMRGULE-LURJTMIESA-N	ESI (-)	168.0773	9.16
	PC P-36:5 or PC O-36:6	-	ESI (+)	764.5588	4.76
	PE O-37:5 (PE O-17:1_20:4)	SUCZWPZZJOWHRI-UHFFFAOYSA-N	ESI (-)	736.5283	6.04
	N-Methylhistidine	CYZKJBZEIFWZSR-LURJTMIESA-N	ESI (+)	170.0911	8.93
	PE P-36:4 or PE O-36:5	ADWDFBQPQIEGRZ-XBICFDGKSA-N	ESI (+)	724.5285	5.33
	PC P-38:6 or PC O-38:7	-	ESI (+)	790.5719	4.97
	PE O-38:6 (PE O-18:1_20:5)	KCNBSSYOJRUKOM-UHFFFAOYSA-N	ESI (-)	748.5292	6.02
	PE 36:4	KZLUVTCXBFEIFJ-XGLJQOENSA-N	ESI (+)	740.5232	5.05
	3-Aminotyrosine	POGSZHUEECCEAP-UHFFFAOYSA-N	ESI (+)	197.0888	7.98
	PE 38:5 (PE 16:0_22:5)	BXSFSSDQHFBPDQ-UHFFFAOYSA-N	ESI (-)	764.5232	5.54
	(2R)-3-Hydroxyisovaleroylcarnitine	IGLHHSKNBDXCEY-SECBINFHSA-N	ESI (+)	262.1631	6.90
	PE 36:1 (PE 18:0_18:1)	JQKOHRZNEOQNJE-UHFFFAOYSA-N	ESI (-)	744.5541	6.83
	PE P-38:3 or PE O-38:4	YHSWSUHZIXJBHZ-OWTAGOBYSA-N	ESI (-)	752.5585	6.80
	PC O-36:3	GDJAIASCRXEGBE-UHFFFAOYSA-N	ESI (+)	770.6038	5.50
	PE P-38:6 or PE O-38:7	WVGALBKSWOUIEZ-XNHMFJFDSA-N	ESI (-)	746.5137	5.63
	PC P-34:1 or PC O-34:2	MBRHHFWRXQYYAN-JEPFLRBFSA-N	ESI (+)	744.5901	5.34
	PC 40:5 Isomer B	LJFKFKIYUJNFPZ-ZLFSCUDPSA-N	ESI (-)	894.6219	6.32

	(3-Carboxypropyl)trimethylammonium	JHPNVNIEXXLNTR-UHFFFAOYSA-O	ESI (+)	146.116	7.53
	PC P-34:1	MBRHHFWRXQYYAN-RTVLTNFHSA-N	ESI (-)	802.5961	5.88
	PC O-37:5	ZOCNILJFNAHQJJ-UHFFFAOYSA-N	ESI (+)	780.5926	5.35
	Genistein	TZBJGXHYKVUXJN-UHFFFAOYSA-N	ESI (+)	271.0598	1.05
	Daidzein	ZQSIJRDFPHDXIC-UHFFFAOYSA-N	ESI (+)	255.063	1.15
	N-α-Acetyl-L-ornithine	JRLGPAXAGHMNOL-LURJTMIESA-N	ESI (+)	175.106	7.95
GC-TOF-MS	Beta-alanine	UCMIRNVEIXFBKS-UHFFFAOYSA-N	-	90.053	7.79

ESI, electrospray ionization; GC-TOF-MS, gas chromatography time-of-flight mass spectrometry; InChI, the IUPAC International Chemical Identifier; PC, phosphatidylcholine; PE, phosphatidylethanolamine; RT, retention time; UHPLC-QTOF MS/MS, ultra-high performance liquid chromatography-quadrupole time-of-flight tandem mass spectrometry.

Supplemental Table S3 Plasma metabolites with variable importance in projection (VIP)[1] scores > 1.0, comparison between animal protein and soybean protein diets

Metabolite	Pathway involved	VIP score[1]
Daidzein 4'-sulfate	Xenobiotics	16.5
Genistein	Xenobiotics	7.22
Daidzein	Xenobiotics	7.16
3-Methylhistidine	Amino acid	4.57
N-α-Acetyl-L-ornithine	Amino acid	2.55
3-Aminotyrosine	Amino acid	2.54
PE O-37:5(PE O-17:1_20:4)	PE/lipid metabolism	2.49
PE P-36:5 or PE O-36:6	PE/lipid metabolism	2.03
PE O-38:6 (PE O-18:1_20:5)	PE/lipid metabolism	1.97
β-alanine	Amino acid	1.73
4-Methylcatechol	Amino acids	1.69
Liquiritigenin	Xenobiotics	1.68
PE P-34:2 or PE O-34:3	PE/lipid metabolism	1.57
4-Pyridoxate	Vitamin	1.52
PC P-36:5 or PC O-36:6	PE/lipid metabolism	1.46
L-Cysteine S-sulfate	Amino acids	1.45
PE P-36:4 or PE O-36:5	PE/lipid metabolism	1.43
Docosahexanoic acid	Lipids	1.42
PE P-40:6 or PE O-40:7	PE/lipid metabolism	1.32
Nudifloramide	Vitamin	1.29
PE P-38:6 or PE O-38:7	PE/lipid metabolism	1.20
TG 55:4|TG 18:1_19:1_18:2	Lipids	1.19
PE P-38:3 or PE O-38:4	PE/lipid metabolism	1.14
PC P-38:6 or PC O-38:7	PE/lipid metabolism	1.13
N-Methylhistidine	Amino acids	1.13

[1]Variable importance in projection (VIP) score was calculated using partial least-squares discrimination analysis. This table lists plasma metabolites with VIP scores greater than 1.0. PC, phosphatidylcholine; PE, phosphatidylethanolamine; TG, triglyceride.

Supplemental Table S4. Cardiometabolic risk factors of the study participants at the end of each 5 week diet phase

Variables	Animal Protein	Soybean Protein
Total cholesterol, mmol/L	5.76 ± 0.53	5.65 ± 0.60
Triacylglycerol, mmol/L	1.27 ± 0.47	1.44 ± 0.70
VLDL-C, mmol/L	0.36 ± 0.24	0.41 ± 0.31
LDL-C, mmol/L	3.80 ± 0.53	3.66 ± 0.44
HDL-C, mmol/L	1.61 ± 0.39	1.58 ± 0.46
HDL_2-C, mmol/L	0.65 ± 0.29	0.62 ± 0.30
HDL_3-C, mmol/L	0.95 ± 0.14	0.96 ± 0.18
TC/HDL-C	3.79 ± 1.01	3.81 ± 1.05

Values are presented as mean±S.D. (N=11). The table was adapted from our previous publication.[24] HDL-C, high-density lipoprotein-cholesterol; LDL-C, low-density lipoprotein-cholesterol; TC/HDL-C, the ratio of total cholesterol: high-density lipoprotein-cholesterol; VLDL-C, very low-density lipoprotein-cholesterol.

Note: There were no significant differences between animal protein and soybean protein diets.

Supplemental Table S5. Pearson Correlation Coefficient between top 10 plasma metabolites and cardiovascular disease risk factors - animal protein diet and soybean protein diet

Plasma metabolite	TC	TG	VLDL	LDL	HDL	HDL_2	ApoA1
				Animal protein diet			
Daidzein 4'-sulfate	-0.346	-0.108	-0.097	-0.268	-0.043	-0.096	-0.020
Genistein	0.113	0.257	0.265	-0.105	0.147	-0.034	0.419
Daidzein	-0.210	-0.106	-0.313	-0.210	0.188	0.299	0.028
N-α-acetyl-L-ornithine	-0.077	0.051	-0.025	-0.139	0.104	0.087	0.039
3-Aminotyrosine	-0.032	0.117	-0.023	-0.098	0.109	0.073	0.095
3-Methylhistidine	0.319	-0.227	-0.081	0.284	0.085	0.305	-0.191
PE O-37:5 (PE O-17:1_20:4)	-0.160	-0.214	-0.282	-0.130	0.122	0.182	0.004
PE O-38:6 (PE O-18:1_20:5)	-0.103	-0.571	-0.528	-0.189	0.433	0.497	0.256
PE P-36:5 or PE O-36:6	0.477	-0.001	0.234	0.316	0.068	0.095	0.011
β-alanine	0.188	0.035	0.198	0.323	-0.317	-0.165	-0.511
				Soybean protein diet			
Daidzein 4'-sulfate	0.265	0.257	0.267	0.253	-0.070	0.026	-0.122
Genistein	0.356	0.212	0.156	0.243	0.133	0.195	0.131
Daidzein	0.345	0.206	0.159	0.265	0.096	0.165	0.090
N-α-acetyl-L-ornithine	-0.132	0.571	0.591	-0.254	-0.326	-0.370	-0.349
3-Aminotyrosine	-0.043	0.521	0.591	-0.223	-0.239	-0.267	-0.288
3-Methylhistidine	-0.095	0.019	0.041	0.074	-0.222	-0.126	-0.359
PE O-37:5 (PE O-17:1_20:4)	0.775^{*}	-0.494	-0.314	0.742^{*}	0.524	0.581	0.564
PE O-38:6 (PE O-18:1_20:5)	0.621^{*}	-0.697^{*}	-0.608^{*}	0.431	0.817^{*}	0.877^{*}	0.781^{*}
PE P-36:5 or PE O-36:6	0.496	-0.597	-0.600	0.437	0.642^{*}	0.712^{*}	0.624^{*}
β-alanine	-0.168	0.016	-0.118	-0.163	0.014	-0.002	-0.006

Values were presented as correlation coefficient (*r*). *, $p<0.05$. ApoA1, apoprotein A1; HDL, high-density lipoprotein; LDL, low-density lipoprotein; TC, total cholesterol; TC, triglyceride

Supplemental Table S6. Composition of the 2 experimental diets

Variables	Animal Protein Diet	Soybean Diet
	% of energy	
Carbohydrate	54.5	57.0
Protein	16.9	14.9
Soybean protein	-	7.5
Arginine: lysine	0.8	1.1
Total fat	28.4	28.0
SFA	7.0	7.3
12:0	0.4	0.5
14:0	0.6	0.7
16:0	3.9	4.1
18:0	1.7	1.5
MUFA	10.3	9.5
16:1	0.2	0.1
18:1	9.1	9.1
PUFA	9.9	10.0
18:2n-6	7.9	8.9
18:3n-3	1.3	1.4
20:4n-6	0.1	0.1
Cholesterol (mg/1000kcal)	83.3	77.3
Fiber (g/1000 kcal)	14.2	16.4
Isoflavones (mg/1000kcal)	10.4	66.0
Daidzein	0.6	15.4
Genistein	9.8	47.9
Glycitein	0.0	2.8
Total plant sterols (mg/1000kcal)	150.8	151.6
Oxalates (g/1000kcal)	0.09	0.09
Folates (mg/1000kcal)	0.11	0.18
Phytates (%)	<0.2	<0.2

Adapted from Matthan et al.[13] The data of carbohydrate, protein, and fat are presented as percentage of total calories, and other compositions are presented as indicated. MUFA, monounsaturated fatty acids; PUFA, polyunsaturated fatty acids; SFA, saturated fatty acids.

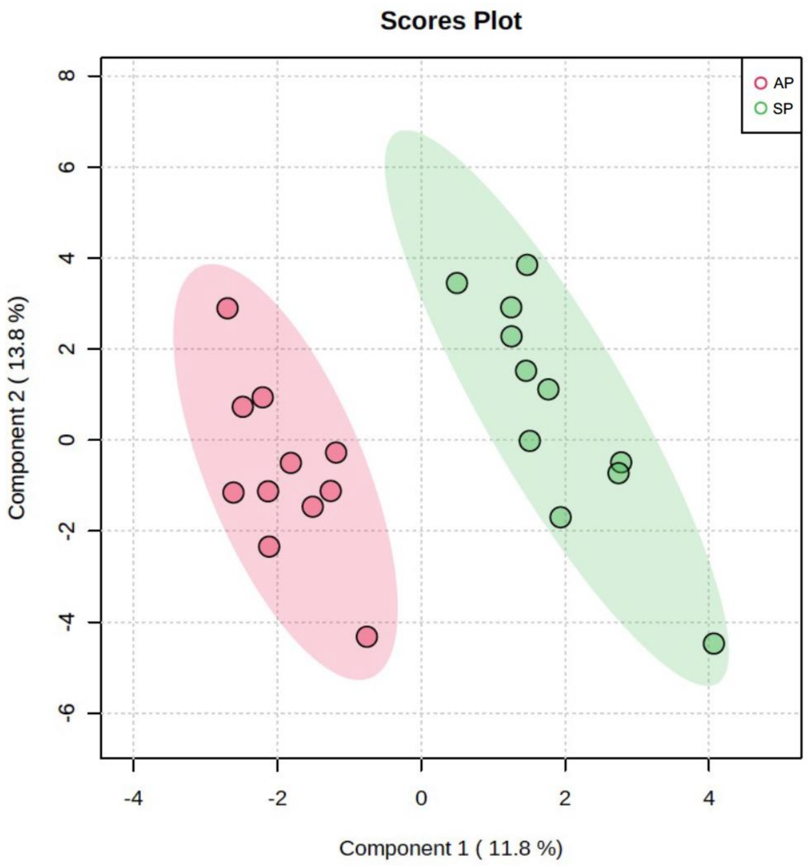

Supplemental Figure S1. Principal component analysis of the plasma metabolites in participants after they received the animal protein enriched diet, compared to the soybean protein enriched diet.

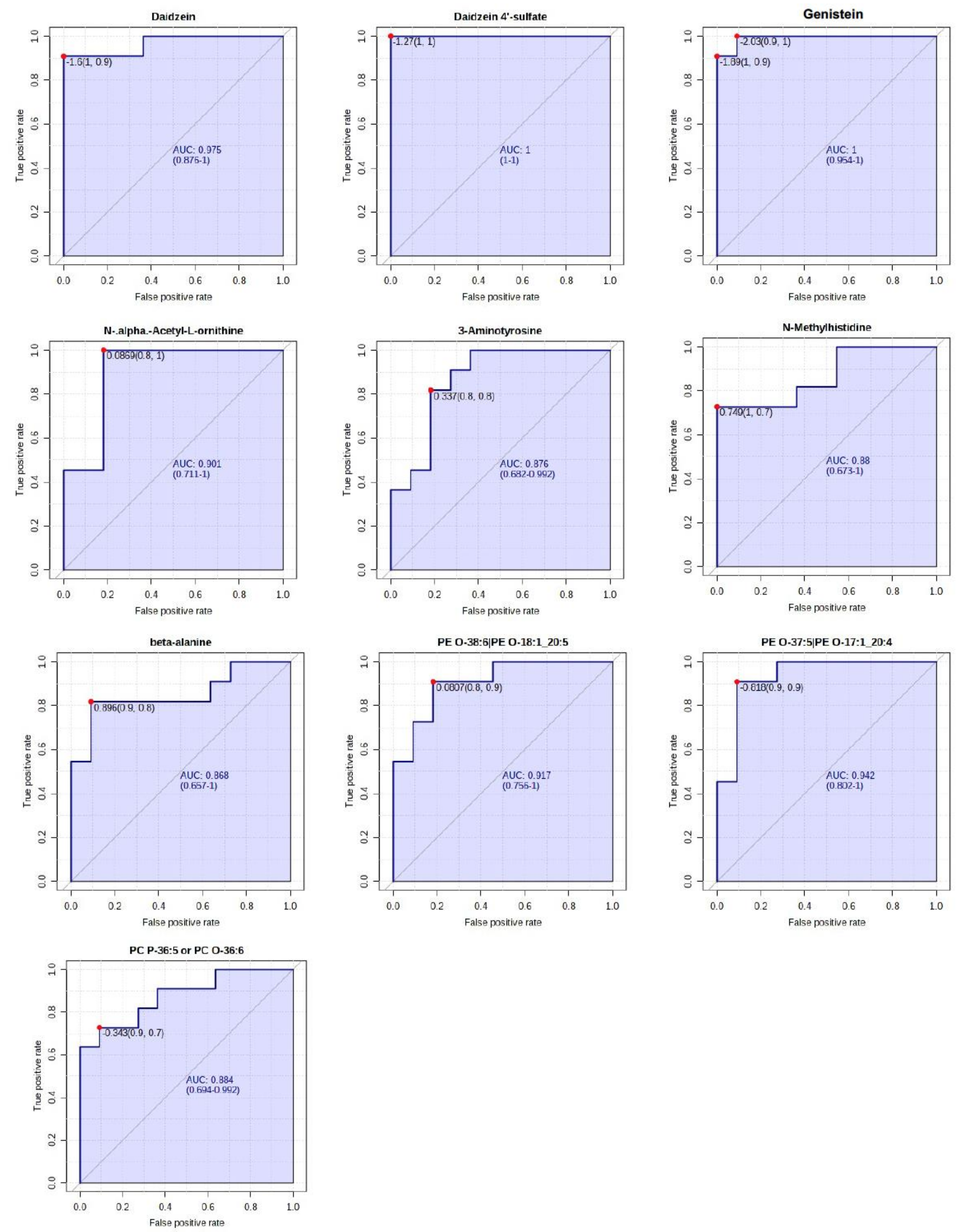

Supplemental Figure S2. Area under the curve-receiver operating characteristics (AUC-ROC) curve for the top 10 plasma metabolites.

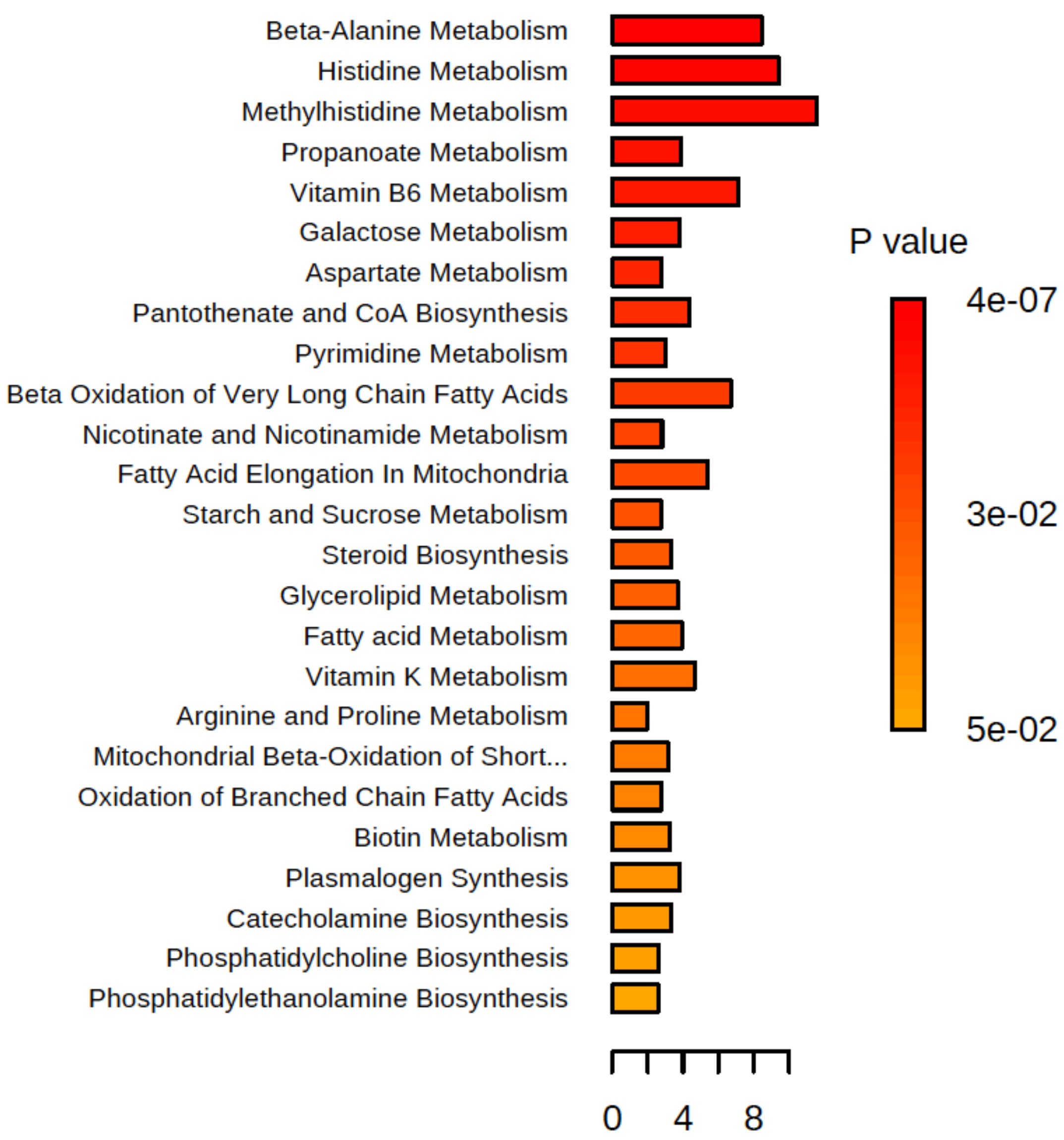

Supplemental Figure S3. Quantitative enrichment analysis of the plasma metabolites in participants after they received the animal protein enriched diet, compared to the soybean protein enriched diet.
